# Mobilomics in *Saccharomyces cerevisiae* strains

**DOI:** 10.1186/1471-2105-14-102

**Published:** 2013-03-20

**Authors:** Giulia Menconi, Giovanni Battaglia, Roberto Grossi, Nadia Pisanti, Roberto Marangoni

**Affiliations:** 1 Istituto Nazionale di Alta Matematica, Città Universitaria, Roma, Italia; 2 Dipartimento di Informatica, Università di Pisa, Pisa, Italia; 3 Dipartimento di Biologia, Università di Pisa, Pisa, Italia; 4, CNR–Istituto di Biofisica, Italia; 5 LIACS – Leiden Institute of Advanced Computer Science, Leiden University, Leiden, the Netherlands

## Abstract

**Background:**

Mobile Genetic Elements (MGEs) are selfish DNA integrated in the genomes. Their detection is mainly based on consensus–like searches by scanning the investigated genome against the sequence of an already identified MGE. Mobilomics aims at discovering all the MGEs in a genome and understanding their dynamic behavior: The data for this kind of investigation can be provided by comparative genomics of closely related organisms. The amount of data thus involved requires a strong computational effort, which should be alleviated.

**Results:**

Our approach proposes to exploit the high similarity among homologous chromosomes of different strains of the same species, following a progressive comparative genomics philosophy. We introduce a software tool based on our new fast algorithm, called regender, which is able to identify the conserved regions between chromosomes. Our case study is represented by a unique recently available dataset of 39 different strains of *S.cerevisiae*, which regender is able to compare in few minutes. By exploring the non–conserved regions, where MGEs are mainly retrotransposons called Tys, and marking the candidate Tys based on their length, we are able to locate *a priori* and automatically all the already known Tys and map all the putative Tys in all the strains. The remaining putative mobile elements (PMEs) emerging from this intra–specific comparison are sharp markers of inter–specific evolution: indeed, many events of non–conservation among different yeast strains correspond to PMEs. A clustering based on the presence/absence of the candidate Tys in the strains suggests an evolutionary interconnection that is very similar to classic phylogenetic trees based on SNPs analysis, even though it is computed without using phylogenetic information.

**Conclusions:**

The case study indicates that the proposed methodology brings two major advantages: (*a*) it does not require any template sequence for the wanted MGEs and (*b*) it can be applied to infer MGEs also for low coverage genomes with unresolved bases, where traditional approaches are largely ineffective.

## Background

Mobile Genetic Elements (MGEs), mostly represented in the eukaryota by transposable elements, are selfish DNA integrated in the genomes. They can vary in length (from hundred up to thousand bases), sequence content, copy number (from a few unities to several thousands) and biological properties from organism to organism (how they replicate and/or jump over the genome and express their own genes). The whole collection of the MGEs hosted in a genome is the *mobilome*[1]. The main relation between MGEs and the host genome is fundamentally parasitic: MGEs tend to replicate by exploiting the resources of the host [2]. By doing this they can destabilize the host organism, as the mutations induced by their jumps or replications can result in gene inactivation or modification. For instance, in the human genome, MGEs are estimated to be around 45% of the total size. Hence, they are a great source of variability and possible diseases, hard to explain by standard inheritance [3,4].

The interaction between MGEs and the host organisms is more complex and still debated in different aspects: apart from the usual parasitic relation, there are also other kinds of interactions, such as direct competition or, at the opposite, cooperation towards synergizing MGEs and their host (see [5] for a detailed discussion on this subject). This scenario suggests to look at genomes, and in particular at eukariotic genomes, as *evolving* ecosystems. Here the *immotile genome* (intended as the complement of the mobilome) and the MGEs act like different species competing for the available biochemical resources [6]–[8].

While population genomists study the mobilome paying particular attention to the dynamics of the MGEs, evolutionary biologists attempt to define the contribution of the mobilome in the evolution of the host organisms. Several studies in evolutively distant organisms suggest that the mobilome has a great impact on the fate of the host. Some evidence supporting this conjecture has been found in all the living kingdom, from prokaryotes [9] to higher eukaryotes [10]–[13] including human [14]. This supports a proposed evolutionary paradigm, where the mobilome drives the evolution of the host organism [15].

The detection of MGEs is mainly based on consensus–like searches, thus scanning the investigated genome against the sequence of an already identified MGE. The identification of new classes of MGEs is a multi–step process that includes chromosomic regions alignments and/or feature detection like testing for the presence of specific repeats and of specific promoters, which depend on the MGEs features in the given organism. We refer to [16] for a recent review of the available tools. Even though these procedures are good at reaching their goals (for example, there can be very fast and still accurate repeat finding tool line that in [17] based on filters ([18])), the whole process can be considered still not efficient, as they cannot guarantee the identification of new classes of MGEs. Experimental procedures can mark the location of MGEs in a genome, also by exploiting high–throughput techniques, but only on the bases of known transposable elements sequences which constitute the probes spotted on the micro–array [19]. We observe that the experimental mapping techniques suffer of the same limitations as the ones described for the other approaches: they need to know the sequence of any class of MGEs, and can give rise to false positive or false negative results on the basis of the similarity between the scanned sequence and the MGE used as a probe.

The importance of having an exhaustive knowledge about the mobilome in an organism motivates the study of this paper. By analogy with other holistic approaches, it is called *mobilomics* and its main goals are: (*a*) providing approaches for a rapid and exhaustive identification of all the mobilome elements in an organism; (*b*) tracking their movements (including replications and deletions) during evolution; (*c*) developing dynamic models able to forecast the fate of the relations between the mobilome and the host genome.

The main contribution of our paper is addressing points (*a*)–(*b*), whereas point (*c*) is to be considered a long–term goal. Note that we already addressed point (*a*) in preliminary work [20], proposing an approach aimed at globally identifying MGEs by an extensive and iterative use of comparative genomics. We expand the preliminary results of [20] and fully discuss point (*a*) in this paper. As for point (*b*), our approach follows the rationale that chromosomal mutations induced by MGEs elements are more frequent with respect to those spanning over segments and uncorrelated with the mobilome [21]. Consequently, we assume as working hypothesis that when we compare very close organisms (e.g. different strains of the same species), the observed chromosomal mutations involving sequences longer than a certain threshold are mainly due to MGE events.

Consider for example the situation in which a strain presents a duplication or a jump of an MGE*e* into a new location. This will interleave the homology of that region with that of another strain where *e* was quiescent. Under this situation, we perform an alignment of homologous chromosomes and mark the non–homologous “island” surrounded by homologous sequences as Putative MGE (hereafter denoted PME) to indicate the possibility of an occurrence of an MGE*e*.

*Mobilomics* comes into play by progressively extending the above alignment to other strains (or even organisms): the set of PMEs becomes more and more populated by all the MGEs that effectively moved or replicated. Clearly, this approach is prone to errors, since we may have both false positives and false negatives. On one hand, an MGE*e* that did not move for that set of organisms, would not be marked this way. On the other hand, a chromosomal mutation uncorrelated with the mobilome could be marked as PME. Nevertheless, our approach exhibits two major advantages with respect to the previous literature. First, it does not require any template sequence for the wanted MGEs. Second, it can be applied also to low coverage genomes with unspecified bases, where traditional approaches are largely ineffective.

We illustrate our approach using a case study represented by a set of 39 strains of the yeast *Saccharomyces cerevisiae*, the genome sequences recently released by [22]. They have a low coverage (one–to–fourfold), and so they are unannotated and rich of *unresolved* regions (i.e. sequences of unspecified bases). To have a referral point, we adopt the S288C strain, called RefSeq hereafter, as it is fully sequenced and annotated in the SGD database [23], along with its MGEs.

Different reasons led us to choose the yeast to perform this study. First, to the best of our knowledge, it is the only organism having so many different strains sequenced, thus allowing us to have a large dataset for our tests. Second, the yeast is probably the most known organism from a molecular point of view. Indeed, RefSeq is accurately annotated: MGEs in RefSeq are almost all LTR–retrotransposons, here called *Ty* (Long Terminal Repeats, i.e., both the edges of the transposable element host repeat sequences of about 300 bp length). As of today, five different families of Ty are reported, appearing in several copies on the 16 chromosomes, the positions of which are annotated [23]. The Ty dynamics is actually more involved: first Tys are copied, then the original template is eventually deleted and the two events are almost simultaneous [24]. Hence, the transposition event in the yeast is not properly a *jump* nor a *movement* but, for the sake of simplicity, we will adopt the latter terms in the rest of the paper, with the above proviso.

It is worth noting that Caspi and Pachter [25] also present an approach based on comparative genomics. However, their ideas and implementation are completely different from ours. In their first phase, Caspi and Pachter cluster genomes into homology regions, building an homology map, and then align only homologous tracts. This homology map is based also on evolutionary considerations (relative conservation of exons with respect to introns, etc.). This is completely missing in our approach when aligning chromosomes. In their second phase, Caspi and Pachter use a multi–alignment tool, while we always proceed via pair–alignment using our software tool REGENDER. In their third phase, Caspi and Pachter interpret the data coming from the multi–alignment using a suitable evolutionary tree as input, onto which they map the results. Even in this step, our methodology is completely different, as we try to infer evolutionary relationships as an outcome, and not to use evolutionary data coming from other sources as input data.

### Approach

The application of progressive comparative genomics to PMEs inference represents an approach driven by data analysis, which has been developed in three main steps.

#### ***First step***

We exploit the high similarity among the genomes of the considered yeast strains, to obtain a rapid and efficient masking of the conserved regions to highlight the non–conserved regions, where any MGE that has actually moved is presumably located. This step led us to write and implement an algorithm, called REGENDER, able to perform the extraction of conserved regions between very large sequences in a fast and efficient way. It is presented in the detail in section “Methods” of this paper, and it is publicly available at [26].

#### ***Second step***

We apply REGENDER to a pairwise comparison of all the 16 chromosomes of RefSeq to their homologous ones in two selected strains, so as to analyze the detected non–conserved regions—how they can be classified and how they relate with MGEs.

#### ***Third step***

We perform a simultaneous masking of all the conserved regions in the 39 strains, and a marking of all the PMEs. By focusing on the complete Ty sequences annotated in RefSeq, we perform a validation of the marked sequences and their effective relation with the mobilome.

Some preliminary results about our data analysis with the proposed algorithm (first two steps) have been presented in a conference [27]. In this paper we extend these results and perform a deeper study of pairwise comparisons to show the complete and *de novo* results for the multiple strain comparison. To our surprise, clustering the binary vectors obtained by marking the presence/absence of candidate MGEs in each of the strains provides an interesting evolutionary relation among the strains: it is quite close to that inferred by classic phylogenetic methods based on SNPs analysis.

## Results and discussion

### Preliminary data analysis

Following the usual notation of the Genome Browser at UCSC [28], we name a chromosome pair (Chr*N*_A_,Chr*N*_B_), where 1 ≤ *N* ≤ 16 is the chromosome number, A is RefSeq and B is either Y55 or YPS128 (both strains of the dataset). We chose the latter two strains as testing samples because of their evolutionary distance and different degree of engineerization in labs (see suppl. mat. in [22]). By defining an *L*–gram as a segment of *L* consecutive bases (e.g. the n–grams in [29]), we examined all the possible (overlapping) *L*–grams of Chr*N*_B_ as candidates.

Note that the *L*–grams thus examined are *m* - *L* + 1 in number, accounting for possible duplicates, where Chr*N*_B_ contains *m* bases. Call *valid* the *L*–grams containing only resolved bases. The *commonL*–grams are the valid *L*–grams that occur *exactly* (i.e. fully conserved with no mutation) both in Chr*N*_A_ and Chr*N*_B_.

When B is Y55, the values of *m* are in the range 248 230…1 522 657; when B is YPS128, the values of *m* are in the range 251 809…1 546 313. In our experiments, *L* = 32 resulted to be a good choice (for a statistical discussion see [20]). The following empirical facts were observed for chromosomes *N* = 2,3,…,16, with chromosome *N* = 1 being an outlier (whose percentages are shown inside parentheses below).

(*a*) The *validL*–grams are numerous: they are between 89.53% – 98.54% in Y55 and between 88.84% – 97.27% in YPS128 (81.32% in Y55 and 77.29% in YPS128 for chromosome 1).

(*b*) The *commonL*–grams are also numerous: they are between 74.02% – 84.35% in Y55 and between 71.71% – 83.24% in YPS128 (59.92% in Y55 and 58.47% in YPS128 for chromosome 1).

(*c*) The common *L*–grams that occurs *once* are the vast majority: indeed, those occurring two or more times are very few, between 0.11% – 2.15% in Y55 and 0.07% – 1.93% in YPS128.

A summary reporting the above percentages for the *L*–grams in the 16 chromosomes of Y55 is shown in (Additional file 1: Table S1). An implication of observations (*a*)– (*c*) is that we can localize the conserved regions using the common *L*–grams.

### Pairwise analysis and PMEs inference

By using REGENDER, we then executed the pairwise comparison on all the 16 chromosomes of the two selected sample strains against RefSeq. REGENDER has proven to be very fast: for instance, it can process the longest pair of chromosomes (Chr4, about 2Mb) in only 6 seconds on a standard machine; the global experiment involving the three strains has required less than 10 minutes. Compared with other existing similar tools, REGENDER turns out to be, on average, from four to ten times faster. More details are provided in Supplementary Material (see Additional file 2).

**Figure 1:**
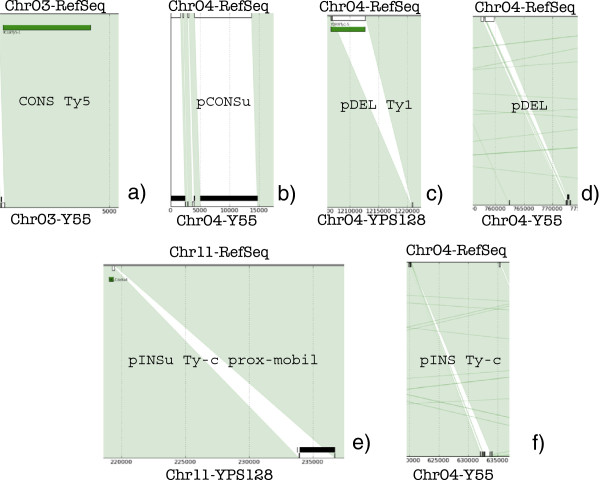
**Categories of features detected after REGENDER results.** In all the sub–figures, the top line represents RefSeq genome while the bottom line represents either Y55 or YPS128. Both the lines are focusing a specific region of a given chromosome, indicated in the labels above the top line and below the bottom one, respectively. The numbers on the bottom line represent the genomic co–ordinate of the considered chromosome. **a**) Conservation: a region annotated as Ty in RefSeq is conserved in the other strain. **b**) Putative conservation: the region of RefSeq corresponds to an unresolved segment, of about the same length, in the other strain. We suppose a conservation. **c**) Putative deletion of a Ty. A region annotated as Ty in RefSeq is missing in the other strain. **d**) Putative deletion of a generic sequence. **e**) Putative insertion of a Ty–compatible sequence. A sequence with length comparable to that of a Ty element is found in the screened strain, while it is apparently missing in RefSeq. The sub–annotation proxy–mobil is due to the existence in RefSeq of an annotation related to MGE very close to the region putatively inserted. **f**) Putative insertion of a sequence Ty–compatible for its length.

A graphical representation of the output of REGENDER is reported in Figure 1, where the top line always represents a region of a chromosome of RefSeq, while the bottom line represents the same region in either Y55 or YPS128; a vertical line is drawn when a common *L*–gram is found between the two chromosomes. MGEs annotated in RefSeq are represented by green rectangles placed just below the top line, while unresolved sequences are represented by black rectangles placed just above the bottom line. Dealing with unresolved sequences represents the true challenge of working with the given dataset: in fact, unresolved sequences are too many to be ignored, and, moreover, they are often linked to MGEs, as it will appear in the following. The overall scenario emerging from REGENDER is that most of the chromosomes are constituted by conserved regions: they are graphically covered by a uniform color zone given by the succession of parallel straight lines connecting identical L–grams. Conserved regions are marked as CONS on Figure 1(a). More than 95% in Y55 and 93% in YPS128 are conserved regions, and they can contain also MGEs: we found one truly conserved Ty per strain and a few number of conserved solo–LTRs.

This uniform coverage can be interrupted when, for example, the screened strain has a long run of unresolved bases. These unresolved sequences are graphically marked by black rectangles. When the lines connecting their flanking regions are all parallel, it is likely that this fragment contains exactly the same sequence as RefSeq. In this case, we have an example of putative conservation, marked as pCONSu, that graphically appears as shown in Figure 1(b). As detailed in the following, often pCONSu regions occur where RefSeq shows annotations relative to MGEs and/or to chromosomic rearrangements hotspots.

Cases in which there is a sequence on RefSeq that has no correspondent on the homologous region of the screened strain are putative deletions. Deletions mainly involve the mobilome. They can occur when an MGE is annotated in RefSeq, in which case they are marked as pDEL–Ty or pDEL–LTR, if they occur for Ty or solo–LTR, respectively. Instead, they are marked as pDEL when this putative deletion is not related to MGEs (Figure 1(c),(d)).

Putative insertions are more difficult to categorize, as the screened strain where they take place are not annotated. If the sequence is resolved, we employ standard alignment tools to search it in RefSeq, trying to detect whether the fragment has actually been moved rather than deleted. On the other hand, when the sequence is unresolved, we can explore only two features. First, we check whether or not the length of the inserted sequence is compatible with either a transposon (when the length of the inserted sequence is ≥4000b) or an solo–LTR (when the length of the inserted sequence is ≤500b). We found that from 40% to 50% of the cases, there is a putative mobilome–proximal insertion. Second, we check whether these insertions take place in a region where an MGE is annotated at a distance less than 200b in RefSeq. We have that the large majority of events are involved with the mobilome. For example, the event marked as “pINSu Ty–c prox–mobil” in Figure 1(e) accounts for an insertion in an unresolved sequence, within such a proximity (in the Chromosome 11) in YPS128 strain with respect to RefSeq. Since this insertion takes place less than 200b away from an solo–LTR annotated in RefSeq, we consider this event as “proximal” to an MGE. This is relevant, since several observations in the literature suggest that Tys prefer to migrate in zones where there are solo–LTRs [30]. Finally, Figure 1(f) shows an event of “pINS Ty–c” since the inserted sequence length is compatible with a transposon.

These cases cover the whole spectrum of the situations we have found in our screening. A complete representation of all the 16 chromosomes in both the strains used for this first screening is available at the link “Plots” in [26].

We now give a detailed discussion on conserved and non–conserved regions. Recall that the latter ones are found as deletions and insertions. Deletions occur when there is a sequence in RefSeq that has no correspondent on the homologous region of the other strain. Insertions are almost point–wise and non–conserved regions in RefSeq to which longer non–conserved regions correspond in the homologous chromosome of the other strain. They are more difficult to categorize because only one strain (RefSeq) is annotated and there can be several unspecified bases inside them. Typically 40–50% of the cases show that an insertion is proximal as well as comparable in length to mobilome.

Table 1 shows the data collected for conserved regions. Different Ty classes are considered: Ty1 and Ty2 are the most represented in the Ty panorama of RefSeq (44 out of 50), while Ty3, Ty4 and Ty5 occur just 2, 3, and 1 times, respectively. Most of conserved regions are part of the resident genome, but not all of them. The fraction of conserved Tys or solo–LTRs within conserved regions contains two possible elements: *(1)* the truly conserved Tys (only one per strain: a frequent Ty1 for YPS128 and a rare Ty5 for Y55) or solo–LTRs (in a relative low number), which are exactly mapped from RefSeq into the other screened strain; *(2)* the putative conservations(pCONSu) of annotated Tys or solo–LTRs, which are mapped into unresolved sequences in the screened strain (and, in this case, a direct attribution is impossible). The pCONSu are always found in the telomeres because the presence of long repeats is a source of noise for the assembly phase. In all cases but one, telomeres do not involve sequences related with MGEs. Concerning pCONSu that are outside the telomeres, the number of unresolved sequences that are located in correspondence or in proximity of MGEs, is greater than 90% for Y55 and around 70% for YPS128. This supports the hypothesis that unresolved regions are often located in correspondence of an MGE annotated in RefSeq.

**Table 1:** RefSeq vs either YPS128 or Y55 : CONSERVATION

**RefSeq vs Y55 strain**											
**CONS**					**pCONSu**						
					**Telomere**	**Non–telomere**					
						IN–mobil				prox–mobil	OUT–mobil
						80.60%				11.94%	7.46%
Ty	1	ltr	191			Ty	16	ltr	38	8	5
Ty1		Ty1ltr	149		32	Ty1	10	Ty1ltr	22		
Ty2		Ty2ltr	2			Ty2	4	Ty2ltr	3		
Ty3		Ty3ltr	18			Ty3	1	Ty3ltr	7		
Ty4		Ty4ltr	19			Ty4	1	Ty4ltr	4		
Ty5	1	Ty5ltr	3			Ty5		Ty5ltr	2		
**RefSeq vs YPS128 strain**											
**CONS**					**pCONSu**						
					**Telomere**	**Non–telomere**					
						IN–mobil				prox–mobil	OUT–mobil
						57.00%				12.00%	31.00%
Ty	1	ltr	195			Ty	20	ltr	37	12	31
Ty1	1	Ty1ltr	154		32	Ty1	10	Ty1ltr	20		
Ty2		Ty2ltr	2			Ty2	6	Ty2ltr	4		
Ty3		Ty3ltr	16			Ty3	1	Ty3ltr	7		
Ty4		Ty4ltr	19			Ty4	2	Ty4ltr	4		
Ty5		Ty5ltr	4			Ty5	1	Ty5ltr	2		

Conserved regions (CONS) and putative conserved regions with unspecified bases (pCONSu) vs annotated Ty and solo–LTR in RefSeq, when pairwise compared either to strain Y55 or strain YPS128.

The pCONSu regions are classified either as telomeric or non–telomeric. Moreover, the non–telomeric regions which are conserved on RefSeq are divided in three classes, depending on their position w.r.t. annotated MGE on RefSeq: pCONSu which are labelled as “IN–mobil” correspond to annotated MGE on RefSeq; pCONSu which are labelled as “prox–mobil” are out of annotated MGE but within a distance of ±200*b* from annotated Ty or solo–LTR; pCONSu which are more distant are labelled as “OUT–mobil”.

Tables 2 and 3 refers to non–conserved regions. Deletions occur very often in correspondence to mobilome annotations and the different classes are deleted similarly in the two strains and uniformly with respect to their global distribution on the genome. Concerning the putative deleted regions (pDELs) in RefSeq that do not correspond to annotated Tys nor to solo–LTRs, say in Y55 strain (they are 4 against more than 90 pDELs corresponding to mobilome annotations): we found that the length of the two regions is compatible with that of a solo–LTR. Inserted regions whose length is compatible with that of a Ty are analogous between the two strains, while Y55 strain shows fewer regions proximal to mobilome and more insertions of intermediate length, with respect to YPS128.

**Table 2:** RefSeq vs either YPS128 Y55: DELETIONS

**RefSeq vs Y55 strain**						
**pDEL vs not Ty–annotated in RefSeq**			**pDEL vs Ty–annotations in RefSeq**			
Ty–c	ltr–c	in–between	Ty	33	ltr	58
0	2	2	Ty1	21	Ty1ltr	31
			Ty2	9	Ty2ltr	7
			Ty3	1	Ty3ltr	13
			Ty4	2	Ty4ltr	6
			Ty5		Ty5ltr	1
**RefSeq vs YPS128 strain**						
**pDEL vs not Ty–annotated in RefSeq**			**pDEL vs Ty–annotations in RefSeq**			
Ty–c	ltr–c	in–between	Ty	29	ltr	55
0	2	0	Ty1	20	Ty1ltr	28
			Ty2	7	Ty2ltr	6
			Ty3	1	Ty3ltr	15
			Ty4	1	Ty4ltr	6
			Ty5		Ty5ltr	

Putative deleted regions (pDEL) vs annotated Ty and solo–LTR and non–annotated regions in RefSeq. Labels “Ty–c” and “LTR–c” refer to putative deleted regions whose lengths are compatible with Ty or solo–LTR lengths. Label “in–between” refers to region lengths which are intermediate between Ty–c and solo–LTR–c.

**Table 3:** RefSeq vs either YPS128 or Y55: INSERTIONS

**RefSeq vs Y55 strain**			
**pINS**			
Ty–c	ltr–c	in–between	prox–mobil
3	16	20	38.46%
**pINSu**			
Ty–c	ltr–c	in–between	prox–mobil
12	3	19	44.12%
**RefSeq vs YPS128 strain**			
**pINS**			
Ty–c	ltr–c	in–between	prox–mobil
3	0	3	50.00%
**pINSu**			
Ty–c	ltr–c	in–between	prox–mobil
13	2	28	51.16%

Putative inserted regions without (pINS) and with unresolved regions (pINSu). Label “prox–mobil” refers to regions in RefSeq where the insertion occurs within ***±***200*b* from Ty or solo–LTR.

### Progressively extending PMEs inference via comparative genomics

Our results show that there is a strong relation between non–conserved sequences and MGEs, thus validating the working hypothesis at the basis of our paper. Nevertheless, our approach can give rise to false positives and false negatives. False positives occur when a chromosomic mutation is erroneously marked as PME. False negatives take place when an MGE falls in a conserved region of the compared genomes (i.e. it is shared by the two strains). To minimize the incidence of false positives, more hypotheses about the length of the chromosomic mutation and on its characteristic (when possible) have to be stated. To rule out possible false negatives, instead, one has to enlarge as much as possible the dataset for a simultaneous comparison of several genomes.

**Figure 2:**
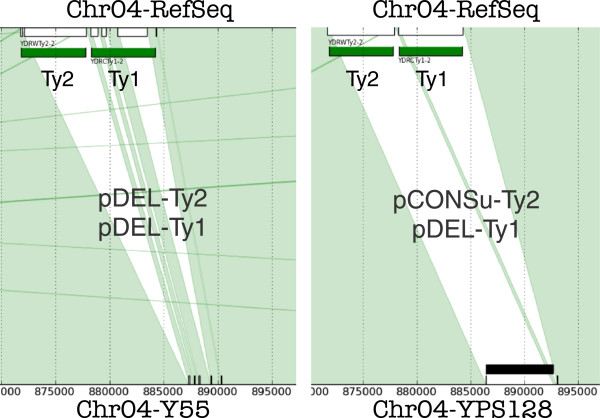
**Populating PME set.** The comparison between RefSeq and YPS128 (right part) finds a putative conservation of a Ty2 element and a putative deletion of a Ty1 element, therefore marked as PME. By means of this comparison alone, the Ty2 has not been recognized as PME. The subsequent comparison between RefSeq and Y55 (left part), where both the Tys have moved, leads to the marking of both these regions as PMEs, thus correctly detecting all the already known Ty elements in this region.

To illustrate this situation with a typical example, let us consider the region within Chr. IV where two Tys (YDRWTy2–2 and YDRCTy1–2) are annotated in RefSeq. Considering again the two strains Y55 and YPS128, we have that the annotated Ty1 is missing in both strains, while the annotated Ty2 is conserved only in YPS128 (see Figure 2). If we considered only YPS128, the mobile nature of Ty2 sequence would have not been noticed: only when considering the comparison to Y55, also Ty2 is labeled PME.

Our progressive addition of strains has populated the PMEs set, and after the comparison of 15 genomes (less than half of the available genome collection), more than 80% of the known Ty in RefSeq have been marked as PMEs, thus showing that the approach is very helpful to recognize MGEs.

Moreover, when implementing pairwise comparisons as in Figure 1(a), an extracted non–conserved region could be marked as PME only by referring to its annotation in RefSeq. Instead, when multiple comparison is performed, a PME can be inferred even if it is conserved in two or more strains: in order to be detected, it is sufficient to find a strain in which it is not conserved. The topology of the resulting multiple alignment, indeed, highlights MGEs *independently of the availability of an annotation*.

This approach allows us to make PMEs inferences also on unannotated and low–coverage genomes, since the putative conservation or deletion may be inferred from the position and the length of the element, disregarding its sequence.

### Mobilomics on 39 strains

With these premises, we analyzed the whole dataset available for our comparison. We run REGENDER simultaneously on the 39 available strains (see section “Methods” subsection “REGENDER on 39 strains” for methodological details), deprived of the telomeric regions (defined as detailed in the Additional file 3: Table S3) because of their intrinsic instability. We marked a large set of PMEs: 649 regions, which vary in their length. To collect information about their actual connection with MGEs we could only refer to RefSeq, the only annotated strain. We therefore mapped on RefSeq all the sequences that are recognized as PMEs, and examined their possible annotations.

To discuss these aspects we focused on two kinds of PMEs, based on their length: those compatible with solo–LTR elements (around 300*b*) and those compatible with a complete Ty element (longer than 4000*b*).

#### ***solo–LTR elements***

The comparison of the PME–LTR candidates and the annotated solo–LTR led to an uncertain situation: only about 44% of the known solo–LTRs are actually marked as putative solo–LTRs. This might have two motivations. First, the large amount of undetected solo–LTRs derives from the low probability that a solo–LTR moves. Indeed, since our approach can recognize only elements that are in non–conserved regions, it means that most of the solo–LTRs are conserved on all the 39 strains. This suggests that it is unlikely that a solo–LTR actually moves. Second, it is not rare to have a chromosomal mutation that spans from 300*b* to 4000*b* on a dataset of 39 strains, and this populates the class of putative solo–LTRs not matching solo–LTR annotations (more than 70%).

Concerning solo–LTR elements, our conclusion is that the comparative genomics approach is ineffective for discovering them, while repeats–finding based approaches perform better.

#### ***Ty elements***

The scenario for PME –Ty candidates is, instead, completely different: REGENDER marks 77 non–conserved regions not shorter than 4000 *b*, that are present in RefSeq.

Focusing on the performance of this kind of Ty–prediction, based on sequence non–conservation, we may say that the test is highly predictive and efficient. The sensitivity $\text{Sn}=\frac{\#\text{True}\;\text{Positives}}{\#\text{True}\;\text{Positives}+\text{\#False}\;\text{Negatives}}$ is 100%, due to the fact that there are no false negative results (all non–telomeric Tys, annotated in RefSeq, have been correctly predicted as non–conserved non–telomeric regions not shorter than 4000 *b*) and the specificity $\text{Sp}=\frac{\#\text{True}\;\text{Negatives}}{\#\text{True}\;\text{Negatives}+\text{\#False}\;\text{Positives}}$ is also high (94,5*%*).

#### ***Genome rearrangement markers and PME –Ty***

We carefully inspected the available experimental annotations on PME –Ty regions, paying a particular attention to those involved on genomic mutations or rearrangements, apart from the MGE annotations already considered. In particular, we considered the following markers, which we indicate as GRms (Genome Rearrangement markers): 

• Autonomously replicating sequences [31] (ARS): They represent the origins of replication in yeast genome.

• Meiotic recombination hotspots [32] (MRhotspot): genomic regions where meiotic recombination double–strand DNA breaks are extremely frequent. They have been associated with high–copy, short–motif microsatellites [33], which play some role in mutation processes in yeast.

• Evolutive and experimental breakpoints [31] (EvolutiveBreakpoint, ExperimentalBreakpoint): evolutionary breakpoints data which are known between *Saccharomyces cerevisiae* and the other yeast *Kluyveromyces waltii*, and between *S. cerevisiae* and a hypothetical ancestor of both yeasts, as well as breakpoints reported in the experimental literature. The two categories are both shown to correlate to early firing origins of replication, contributing to genome rearrangement events.

• tRNA genes [23]: there is a close association between Ty elements and tRNA genes (around 90% of the Ty insertions belonging to the four classes Ty1–Ty4 are found near the tRNAs).

• *γ*– *H*2*A*–rich loci [34]: high–resolution mapping of loci showing accumulation Phosphorylation of histone *H*2*AX*, which is an early response to DNA damage in eukaryotes and are candidate fragile loci.

• Replication termination loci [35](TER): 71 chromosomal termination regions where replication forks stall and which express DNA fragility during cell division. The existence of an evolutionary pressure against TER–containing pause sites on both strands is suggested, perhaps to avoid genome instability events.

The complete list of annotations of genomic features for each PME–Ty marked region is reported in the (Additional file 4: Table S4A and S4B). As a general result, we globally remark that only one region shorter than 5 *kb* contains a full–length Ty association (i.e. a Ty complete of its flanking LTRs), while 35 regions are full–length–annotated out of 46 regions with length above 5 *kb* and under 10 *kb*. Finally, 8 regions have annotated full–length, and 2 of them have pairs of inverted Tys (two adjacent full–length on opposite strands) out of 19 longer regions (longest one is around 32.2 *kb*).

We found that 2 regions did not host any feature. Out of the remaining 75 regions, 44 hosted at least one full–length Ty annotation, 12 at least one solo–LTR annotation, and 19 host some of the above GRms, different from Ty and solo–LTR. Many regions (31) do not involve active MGEs but correspond to loci prone to chromosomic recombination, rearrangement or fragility. We remark that all the known Tys have been correctly marked as PMEs: the only Ty not recognized, is the unique copy of Ty5 that appears in the telomere of Chromosome III, and that has been ruled out from this investigation because of its localization.

Some comments on individual GRms are in order. 

• We notice that 4 out of 6 TERs associated with pme–Tys (TER702, 801, 1601, and 1602) contain two divergent Pol III–dependent pause sites (tRNA/solo–ltr), one of which is proved to be totally or partially not conserved also in other yeast species [35].

• There are 11 regions associated with evolutive breakpoints, which are relative to speciation events.

• Out of 34 regions associated with tRNA genes, only one region does not correspond to annotated full–length Tys nor solo–ltrs.

• ARS–associated regions are likely to contain full–length Tys (35 out of 55).

#### ***Similarity among PME –Tys***

**Figure 3:**
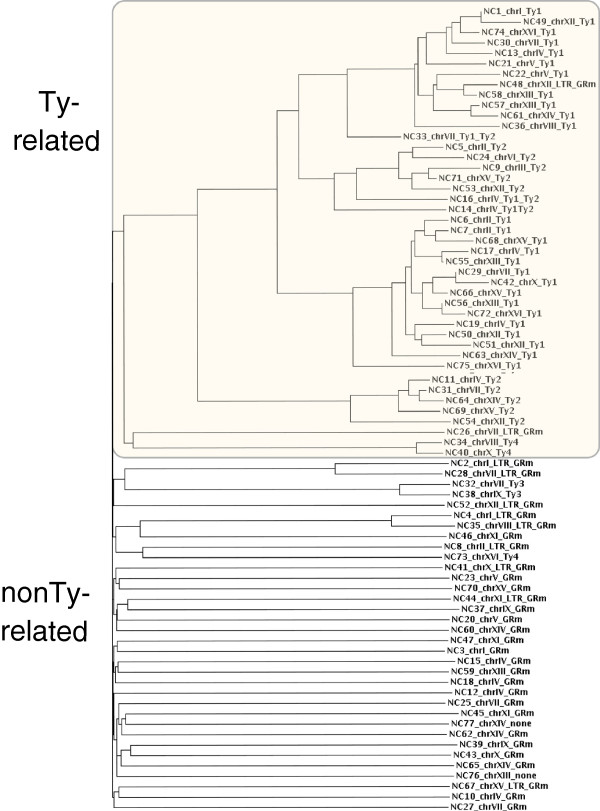
**Phylogram of the 77 PME Ty–candidates sequences.** All the Ty–candidates have been screened for their relative similarity, by means of ClustalW. There is a clear subdivision into two groups: one composed almost all of known Ty, and the other composed almost all of non–Ty, with the exception of the sequences annotated as Ty3.

To deeply screen the possible similarity among these 77 PME Ty–candidates, we downloaded all these sequences from the SGD database. Then we run ClustalW to obtain an essay of their relative similarity. The obtained phylogram shown in Figure 3, clearly cluster the 77 input sequences into two main groups: one composed almost by Ty–annotated sequences, while the other is composed almost all by non–Ty sequences. It is very interesting to inspect the average distance among sub–groups in these two clusters: graphically it is represented by the length of the arcs. The cluster of Ty–sequences shows an inner high relative similarity, since the arcs connecting the sub–groups are short. In the second cluster, instead, the arcs cover all the distance between the sequence and the root, thus showing an inconsistent similarity between the sequences. The only exception is represented by the sequences annotated as Ty3, which shows a slightly more robust relative similarity. This phylogram analysis suggests that non–Ty sequences are really different from each other, and therefore they are unlikely to derive from the movements and/or duplication of a given transposable element.

#### ***Statistics of PME –Ty moves***

We inspected the frequency of movements, by building a Boolean matrix (contained in Additional file 5) on a length–basis: for each PME Ty–candidate *c* and for each yeast strain *s*, we report 1 if *c* is present in *s*, and 0 otherwise.

Summing the 1 values, we obtain a score between 1 and 39. If we sort the PME–Ty list accordingly to these values, we have a clear view on which non–conserved regions are still present in which strains. We can sharply divide the 77 PME–Ty regions into two groups: those length–conserved in 39 strains (42 regions, called almost–conserved) and those conserved in a lower number of strains (35 regions, called fully non–conserved).

We observe that 28 out of 44 sequences corresponding to annotated Tys are fully non–conserved since they score less than 39 (i.e., there is at least a strain where the element is missing), while only 7 out of 33 of the non–Ty (either GRm or also solo–LTR or no association) annotated PME–Ty are fully non–conserved. In other words, while REGENDER identified non–conserved regions w.r.t. sequence conservation, these regions have not necessarily moved in all the examined strains. There are only 16 Ty–annotated PME sequences that appear to maintain their position, possibly with a change in their sequence, across the genomes, but the large disequilibrium between the frequencies of jumps allows us to say that false positive sequences tend to be resident. Out of 11 regions associated with evolutive breakpoints (inter–specific), 8 are length–conserved in all 39 strains, thus supporting the claim that length–conservation is not a primary (intra–specific) event in mobilomics.

To sum up, if we use the length–conservation to distinguish PME–Tys among true annotated MGEs (PME–Tys which are fully non–conserved) and non–MGEs (PME–Tys which are almost–conserved), we get a sensitivity *Sn* = 63.6*%* and a specificity *Sp* = 78.8*%*: this again is a good test to classify mobilomics events.

#### ***Mobilome tree***

**Figure 4:**
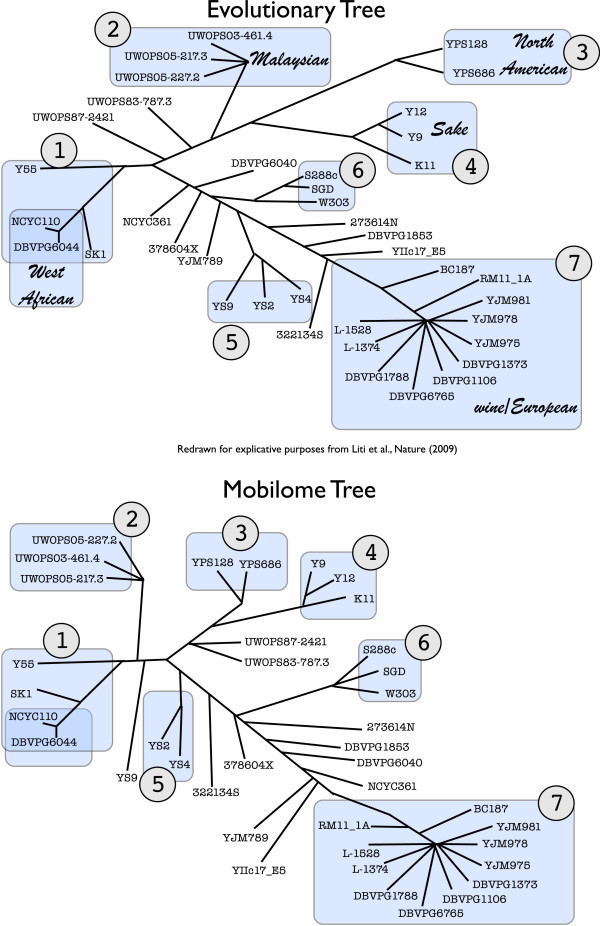
**Evolutionary Tree and Mobilome Tree.** (top) Evolutionary tree (redrawn from [22]) and (bottom) mobilome tree. The latter was created rooted by UPGMA, then redrawn unrooted to be compared to evolutionary tree.

The fact that different sequences marked as PME Ty–candidates have a different degree of presence in the different strains suggested us to try to understand the dynamics of the marked movements. By using the Boolean vectors described above we generated a tree, which we call *mobilome tree*, obtained by scoring the distance between every pair of strains by means of Hamming distance and clustering by UPGMA. Although the obtained mobilome tree is not a phylogenetic tree, it reveals the clusters among strains obtained by minimizing the movements of PMEs. It is really interesting to compare such a non–standard tree with the phylogenetic tree obtained by standard phylogenetic approaches in [22] that are based on SNPs comparison on a set of suitably identified genes. Figure 4 shows this comparison: here the mobilome tree is shown on the bottom, and the phylogenetic tree is shown on the top. Surprisingly, most of the clades determined by following the two independent methods coincide, and this probably represents a further support of the recently established paradigm that Tys are able to drive the evolution of organisms, as reported in [15]. Since a subset of 35 regions are enough to map evolutive clades, it appears that the movement of a single MGE in the genome of a strain is enough to address the strain’s evolution. However, this very strong hypothesis needs further evidences to be evaluated. The most remarkable fact is that the information amount needed for our approach is really minimal, and almost all obtained *a priori*. An interesting side observation is that this picture does not change when annotating the presence/absence of solo–LTR elements as well (that is, setting solo–LTR–c threshold in phase 2 of subsection “REGENDER on 39 strains”). Also in this case the large majority of clades are identical to the classic phylogenetic tree.

## Conclusions

In this paper we proposed an original approach to extend the comparative genomics employed to discover mobile genetic elements. We released a software tool able to perform the required computations in an efficient and powerful way. We applied this approach to the recent available dataset of 39 genomes of the yeast, where we proved that the approach is able to correctly identify the already known Ty elements, with no false negatives. About possible false positives, we showed that they are non–conserved regions very unlikely to move and, by extending the approach, probably they will be discriminated from true MGEs. We also showed that the PME presence/absence seems to parallel the evolutionary history of the yeast without relying on evolutionary data coming from other sources as input data. Our case study shows that the method can be applied to infer MGEs also for large data sets of low–coverage genomes with unresolved bases, where traditional approaches are largely ineffective. A promising avenue is to dig into the data streams arising from NGS.

Future work is to extend the proposed approach to inter–specific comparisons, where the underlying hypothesis that most of the longest chromosomal mutations are due to MGEs should be made weaker.

## Methods

### Preliminary data analysis

#### ***Approach***

##### 

**Conserved regions** Our algorithm for the rapid detection of large highly–conserved segments, called REGENDER (REsident GENome DEtectoR), performs a two–phase processing of all the possible chromosomes’ pairs (Chr*N*_A_,Chr*N*_B_), where A is RefSeq, B is either Y55 or YPS128, and *N* ranges from 1 to 16. In the first phase, REGENDER finds the common *L*–grams between Chr*N*_A_ and Chr*N*_B_. In the second phase, REGENDER aggregates consecutive *L*–grams in a greedy fashion using some user–defined parameters that control when the next conserved region begins in both Chr*N*_A_ and Chr*N*_B_.

REGENDER is somewhat related to the *anchor–based* algorithms [36] that circumvent the quadratic cost (time and space) of the textbook algorithms for sequence alignment (e.g. [37]). This family is quite populated since large–scale genome comparison is time– and space–demanding: WABA[38], BLASTZ[39], PIPMAKER[40,41], BLAT[42], ASSIRC[43], GLASS[44], LSH-ALL-PAIRS[45], and PATTERN-HUNTER[46,47], to name a few. Other similar approaches are described in [48]–[51].

The above algorithms share a common mechanism. First, they build a dictionary (e.g. hash table, trie, or automaton) to store the fragments or seeds (e.g. the *L*–grams) that are common to both Chr*N*_A_ and Chr*N*_B_. Second, they extend the fragments/seeds into longer sequences called *anchors* using dynamic programming (except chaining algorithms by [36]). The sequence of anchors thus found are required to be *colinear*; namely, the anchors should occur in the same relative order inside both Chr*N*_A_ and Chr*N*_B_. Third, these algorithms apply an expensive dynamic programming scheme to the regions of Chr*N*_A_ and Chr*N*_B_ that are left uncovered by the anchors.

REGENDER can go simpler. First, the *L*–grams of Chr*N*_A_ can be stored in a hash table, and those of Chr*N*_B_ can be searched in the table during a scan of Chr*N*_B_. The high similarity of Chr*N*_A_ and Chr*N*_B_ justifies our choice of exact *L*–grams as fragments. Recall that Chr*N*_A_ and Chr*N*_B_ are the *same* chromosome of two different strains of *S.cerevisiae*.

Second, our dataset gives almost surprisingly a natural set of anchors: contrarily to the anchor–based algorithms, we do not need any dynamic programming or chaining techniques to enforce the colinearity and the non–overlapping property, since there is almost a one–to–one mapping between the occurrences of the *L*–grams. Actually, we take advantage of the fact the *L*–grams overlap and, if they are not colinear, we get a hint for a possible translocation.

**Figure 5:**
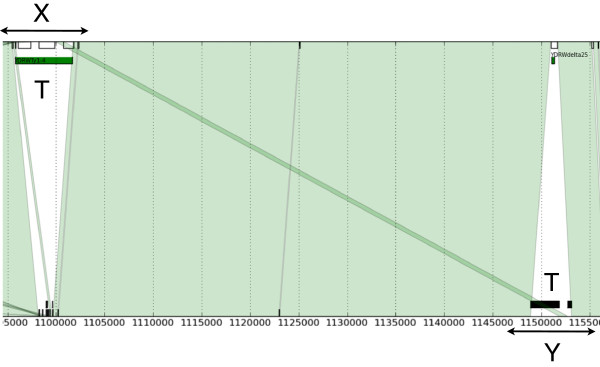
**REGENDER graphical output.** A fragment of the plot of the common *L*–grams for Chr4 (1 095 000–1 155 000) of RefSeq (top sequence) and Y55 (bottom sequence), where *L* = 32. Each line connects the starting positions of a common *L*–gram. The white triangles or trapezoids thus highlight non–conserved regions. Annotated mobile elements are represented by green rectangles placed just below the top line; unresolved sequences are represented by black rectangles placed just above the bottom line.

A visual inspection of Figure 5 can confirm this fact, where a line connects the starting position of two *L*–grams, one in Chr*N*_A_ and the other Chr*N*_B_, when they match. We can observe that our dataset generates very few line intersections. Also, the non–conserved regions are singled out as “empty triangles or trapezoids.”

As a result, REGENDER performs just a scan of Chr*N*_A_ and Chr*N*_B_. One execution of REGENDER takes few seconds on a standard PC with limited amount of memory. This is a major requirement, since we need to execute REGENDER for all pairs of corresponding chromosomes of Chr*N*_A_ and Chr*N*_B_.

Third, we remark that we do not need a complete alignment of Chr*N*_A_ or Chr*N*_B_ for the purposes of the analysis performed in this paper. A high–quality alignment of the conserved regions in Chr*N*_A_ or Chr*N*_B_ is unnecessary in our case, as illustrated by the clear patterns emerging from Figure 5. What we really care about is the description of the dynamics of the mobilome, identifying and locating all the MGEs in the input sequences, together with the genomic rearrangements they are involved into. A merit of our approach is that of being able to select a small set of candidates for the latter investigation, as discussed next.

##### 

**Non–conserved regions** The outcomes of our experiments with REGENDER are analyzed as follows.

Graphically, we represent the two homologous chromosomes as two horizontal straight lines, and place A in the top and B in the bottom, as in Figure 5. We mark the conservations with some color. The non–conserved regions are then detectable as non–coloured trapezoids. The action of a transposable element *T* that has changed position from region *X* of strain A to region *Y* of strain B within two homologous chromosomes is then represented by two triangles (Figure 5): we detect a white downward triangle inside region *X* (marking presence of *T* only in region *X* of strain A and absence in strain B), and an upward white triangle in *Y* (marking presence of *T* only in region *Y* of strain B and absence in corresponding position on strain A). Therefore, when strain A is RefSeq, we can infer that *T* probably moved from *X* to *Y* inside strain B by projecting region *X* of A onto the corresponding part in B. A picture of possible situations is shown with some detail in Figure 1.

We followed the above conceptual scheme to collect statistics for all the chromosomal rearrangements among the 16 chromosomes’ pairs from the selected strains (B is Y55 or YPS128) with the same chromosome in A=RefSeq, thus classifying any resulting rearrangement. We refer the reader to section “*Results and discussion*” for an aggregate view of all the chromosomal differences found and their relation with the mobilome. We remark that we considered significant events that involve regions containing at least 200b, since very short indels or mutations are not linked with mobilome nor with chromosomal rearrangements.

The proposed approach allowed us to obtain a fast and efficient localization of the resident genome, by working on a standard computer. Our results clearly show that the significant chromosomal indels involve almost exclusively the mobilome. Moreover, we show that unresolved sequences take place almost always in the correspondence of telomeres or MGEs. Our approach allows us to infer putative insertions and deletions of transposons or solo–LTR elements also in the presence of unresolved sequences.

## Algorithm and implementation

As previously mentioned, we exploit the high similarity between genomes of different strains by running a massive computation involving all the possible chromosomes pairs (Chr*N*_A_,Chr*N*_B_), where A is RefSeq, B is either Y55 or YPS128 strain, and *N* = 1,…,16. We recall that REGENDER follows a two–phase approach, where the inputs are two chromosomes Chr*N*_A_ and Chr*N*_B_, the length *L* of the grams, and two user–defined parameters *δ*_1_ and *δ*_2_ to be used in the second phase. First, it finds all the common *L*–grams between Chr*N*_A_ and Chr*N*_B_. Second, it detects highly conserved regions by aggregating consecutive *L*–grams. Then, we can inspect the non–conserved regions that are found by REGENDER, so as to infer mobilome elements.

### REGENDER on two strains

#### ***Phase 1 of REGENDER: common L–grams***

We aim at finding which *L*–grams of Chr*N*_B_ occur inside Chr*N*_A_, where an *L*–gram is any sequence of *L* consecutive bases. First, we construct a dictionary for all the *L*–grams in Chr*N*_A_ and, then, we search for the *L*–grams of Chr*N*_B_ inside the dictionary. This task can be performed in expected linear time by employing a rolling hash approach based on cyclic polynomial, as described in [52]. Note that using a general purpose hash function would be more expensive by a multiplicative factor of *L*. Also, using a trie–based dictionary instead of hashing would guarantee a linear–time worst–case performance, but hashing is faster in practice.

A detailed description of the rolling hashing is beyond the scope of the current paper. However, the main idea behind this approach is simple. Let assume that each of the four bases, say *c*, is mapped into a 32–bit integer *h*_*c*_. Moreover, let us denote the bit–wise exclusive or by ⊕. Let *s*(-) be the cyclic binary rotation function, which shifts the input bit string to the left, moving the leftmost bit in the rightmost position. For example, *s*(10110) = 01101. We use *s*^*i*^(-) to indicate *s*(-) iterated *i* times on the input value. For example, *s*^2^(10110) = *s*(01101) = 11010.

Given the input *L*–gram *t* = *t*[1]*t*[2]⋯*t*[*L*], its hash value is *h*(*t*) = *s*^*L*-1^(*h*_*t*[1]_)⊕*s*^*L*-2^(*h*_*t*[2]_)⊕…⊕*s*(*h*_*t*[*L*-1]_)⊕*h*_*t*[*L*]_. The resulting value is represented by a 32–bits integer. Computing the hash values in a rolling fashion is done as follows. Suppose *t*^′^ = *t*[2]…*t*[*L*+1] is the *L*–gram following *t*. To quickly compute *h*(*t*^′^) from *h*(*t*), we only need to remove the base *t*[1] and add the new base *t*[*L* + 1]. First, the previous hash value is rotated one position to the left, obtaining *h*^″^ = *s*(*h*(*t*)). Then, the new hash value is *h*(*t*^′^) = *h*^″^⊕*s*^*L*^(*h*_*t*[1]_)⊕*h*_*t*[*L* + 1]_.

Some care is required in handling “unresolved” bases, denoted by N, in the input chromosomes. Since the rolling hash approach cannot handle them, when moving the sliding window of length *L* from left to right, we consider the maximal runs of consecutive bases different from N, provided that they are of length at least *L* (otherwise, they cannot contain any valid *L*–gram inside). In this way, we can amortize the OL initialization cost for the rolling hash, with the run length. The linear average–case cost justifies our choice of the rolling hash approach. In fact, assuming that the lookup operation takes constant time, the cost to create the hash table becomes predominant in the time complexity.

##### 

**Lemma 1.** *The first phase of the algorithm *REGENDER *requires *$O|\text{Chr}N_{\text{A}}|+|\text{Chr}N_{\text{B}}|$*time on average*.

The output of the first phase is a mapping *M*, associating each *L*–gram *s*_2_ of Chr*N*_B_, with its occurrence list *occs*(*s*_2_) in Chr*N*_A_. If *s*_2_ does not occur in Chr*N*_A_, *occs*(*s*_2_) is empty. Although not optimal in the worst case, our hash based approach turned out to be effective on our datasets, yielding few collisions, and allowing us to compare two entire chromosomes in few seconds. We implemented a prototype in Java, using the fastutil Java collections library to reduce as much as possible the memory usage ([53]). The experiments have been performed on an Intel Core 2 Duo 5500 notebook, with 2GB of RAM. The code is single–threaded, and the maximum amount of RAM available for the first phase has been set to 200MB. The value of the parameter *L* has been set to 32, and the load factor of the hash table is set to *α* = 0.75.

#### ***Phase 2 of REGENDER: conserved regions***

During the second phase, the information about the *L*–gram occurrences, stored in the mapping *M* computed in the first phase, is used to establish a correspondence between segments of consecutive bases in Chr*N*_B_ and Chr*N*_A_, mapping a segment *I*_2_ = Chr*N*_B_[*l*_2_,*r*_2_] into a corresponding segment *I*_1_ = Chr*N*_A_[*l*_1_,*r*_1_]. This information is represented by the mapping *M*_2_, and it is graphically shown with green lines in Figure 5.

We perform a left–to–right scan of Chr*N*_A_ and Chr*N*_B_, according to the following greedy rule. Initially, *I*_1_ and *I*_2_ are empty. During the scan, the current segments *I*_1_ and *I*_2_ are extended when the following conditions are met: (*a*) there exists a common *L*–gram *s*, which occurs both to the right of *I*_1_ and *I*_2_, and no other *L*–gram with this property can be found between *I*_1_ and *s*, and *I*_2_ and *s*; (*b*) letting *d*_1_ be the number of bases between *I*_1_ and *s*, and *d*_2_ be the number of bases between *I*_2_ and *s*, it is |*d*_1_ - *d*_2_| ≤ *δ*_2_ and *d*_2_ ≤ *δ*_1_ (hence, *d*_1_ ≤ *δ*_1_ + *δ*_2_). To describe the main steps, assume that the first *j* - 1 bases of Chr*N*_B_ have already been processed, and that $M_{2}^{\prime }$ is the mapping constructed so far. To add the next pair of intervals to $M_{2}^{\prime }$, the main steps are as follows: 

(1) *Starting point search*. The starting point of the next segment is set to the coordinate of the leftmost *L*–gram (say *j*_1_) that does not belong to any previously mapped interval in $M_{2}^{\prime }$, and that occurs at least once in Chr*N*_A_ (i.e. *M*(Chr*N*_B_[*j*_1_…*j*_1_ + *L* - 1]) ≠ *∅*). Let *L*_1_ = {*i*_1_,…,*i*_*p*_} be the nonempty occurrence list *occs*(*s*_2_) in Chr*N*_A_, where *s*_2_ = Chr*N*_B_[*j*_1_…*j*_1_ + *L* - 1]. Among all the identical *L*–grams in *L*_1_, we map *s*_2_ into the nearest one. Namely, we select $i^{*}={\text{argmin}}_{i\in L_{1}}\{|j_{1}-i|\}$. Note that *L*_1_ is a singleton list in the majority of cases in our dataset. In the rest of the current section, *i*^∗^ will be referred as the *image* of *j*_1_. If *s*_2_ and its corresponding occurrence at coordinate *i*^∗^ of Chr*N*_A_ cannot be found, all the segments have been already reported, and the mapping $M_{2}^{\prime }$ is returned.

(2) *Segment extension*. Once a starting point *j*_1_ together with its image *i*^∗^ has been selected, the first *L*–gram *s*_2_ = Chr*N*_B_[*j*_1_…*j*_1_ + *L* - 1] is added to the new segment. At this point, the next *L*–gram $s_{2}^{\prime }=\text{Chr}N_{\text{B}}[j_{2}\ldots j_{2}+L-1]$ is examined, along with its occurrence list *L*_2_ = {*k*_1_,…,*k*_*l*_} mapped by *M*. An occurrence *k*^∗^ that satisfies the following conditions is selected from *L*_2_. First, the maximum number of bases between *s*_2_ and $s_{2}^{\prime }$, must be less than or equal to the user–defined threshold *δ*_1_. In other words, it must be *d*_2_ ≤ *δ*_1_ where *d*_2_ = *j*_2_ - *j*_1_ - *L*. Second, since *s*_2_ precedes $s_{2}^{\prime }$ in Chr*N*_B_, we require that the image of *s*_2_ in Chr*N*_A_, namely *s*_1_  Chr*N*_A_[*i*^∗^…*i*^∗^ + *L* - 1], precedes the image of $s_{2}^{\prime }$ in Chr*N*_A_, $s_{1}^{\prime }=\text{Chr}N_{\text{A}}[k^{*}\ldots k^{*}+L-1]$. Hence, we require that *i*^∗^ < *k*^∗^. Finally, we aim at mapping two *L*–grams that occur closely into Chr*N*_B_, into *L*–grams occurring closely in Chr*N*_A_. We constraint the difference of their distance to be within the user–defined threshold *δ*_2_: it must be |*d*_1_ - *d*_2_| ≤ *δ*_2_, where *d*_2_ = *j*_2_ - *j*_1_ - *L*, and *d*_1_ = *k*^∗^ - *i*^∗^ - *L*. If an occurrence of $s_{2}^{\prime }$ satisfying the above conditions is found, the *L*–gram $s_{2}^{\prime }$ is added as an extension to the current segment. The above steps are repeated to find a new *L*–gram following $s_{2}^{\prime }$ in Chr*N*_B_, and satisfying the above conditions. On the other hand, if $s_{2}^{\prime }$ does not satisfy the above conditions, then the next *L*–gram, $s_{2}^{\prime \prime }$, mapped by *M* into a nonempty occurrence list is selected, and an occurrence satisfying the above conditions is looked for. If such an *L*–gram cannot be found, the extension phase terminates.

(3) *Mapping update*. Let *s*_2_ = Chr*N*_B_[*j*_1_…*j*_1_ + *L* - 1] and $s_{2}^{\prime }=\text{Chr}N_{\text{B}}[j_{2}\ldots j_{2}+L-1]$ be the first and the last *L*–gram of the current segment, and *s*_1_ = Chr*N*_B_[*j*^∗^…*j*^∗^ + *L* - 1] and $s_{1}^{\prime }=\text{Chr}N_{\text{B}}[k^{*}\ldots k^{*}+L-1]$ be their corresponding occurrences selected in the previous two steps (where it can be *s*_1_ = *s*_2_.) The current mapping $M_{2}^{\prime }$ is updated by adding the correspondence between segments Chr*N*_B_[*j*_1_,*j*_2_ + *L* - 1] and Chr*N*_A_[*i*^∗^,*k*^∗^ + *L* - 1].

Steps (1)–(3) are repeated until a new segment is found. At the end, the whole mapping *M*_2_ for the conserved regions (anchors) is returned.

To compute the time complexity of the second phase of REGENDER algorithm, we observe that the sum of the sizes of the occurrence lists in *M* is upper bounded by |Chr*N*_A_| - *L* + 1. In other words, the size of the mapping *M* is $O|\text{Chr}N_{\text{A}}|+|\text{Chr}N_{\text{B}}|$. Steps (1)–(3) can be implemented by a left–to–right scan of the chromosomes.

##### 

**Theorem 2.** *Algorithm *REGENDER*requires*$O|\text{Chr}N_{\text{A}}|+|\text{Chr}N_{\text{B}}|$*time on average*.

#### ***Inspection of non–conserved regions***

The contribution of REGENDER is that of reducing a potentially huge number of candidates to very few of them, so that the direct inspection of the non–conserved regions is doable. We perform this crucial analysis of the regions that have not been mapped into segments by *M*_2_. These are the potential candidates for being mobile elements. We refer the reader to section “*Results and discussion*” for a detailed discussion of the analysis performed.

### REGENDER on 39 strains

Given the 39 homologous chromosomes of the *S. cerevisiae* strains Chr*N*_1_,…,Chr*N*_*k*_,…,Chr*N*_39_ for as many strains *A*_1_,…,*A*_39_ (where *A*_1_ is RefSeq), our goal is to cluster them according to the topology of their mobile elements. This goal is achieved in three phases. 

• Phase 1: applying regender to multiple strains. Given the high computational cost of multiple alignment, and the presence in all the input sequences (except RefSeq) of unresolved bases, we used the RefSeq chromosome as a reference to align all the others. Once the segment–based pairwise alignments between RefSeq and each other input chromosome have been computed, we only report the segments that are conserved in *all* the input chromosomes, by intersecting the conserved segments.

• Phase 2: from sequences to binary vectors. Once we know the conserved segments, let *p*_*N*_ denote the number of non–conserved segments within Chr*N*. Let $S_{k}(N)=(\text{Chr}N_{k}[i_{1},j_{1}],\ldots ,\text{Chr}N_{k}[i_{p},j_{p_{N}}])$ be the left to right sequence of the non–conserved segments in chromosome N of the *k*–th strain. We construct a binary vector *Ŝ*_*k*_(*N*) of the same size as *S*_*k*_(*N*), where the *n*–th component is ^′^0^′^ if the segment Chr*N*_*k*_[*i*_*n*_,*j*_*n*_] is smaller than the user–supplied size threshold *d*, and ^′^1^′^ otherwise. We shall use default thresholds: *d* = 4000 (called Ty–c threshold) and *d* = 300 (called solo–ltr–c threshold) according to whether we want to detect transpons only or also fragments as short as solo–ltrs, respectively. Let *Ŝ*_*k*_ = *Ŝ*_*k*_(1)*Ŝ*_*k*_(2)⋯*Ŝ*_*k*_(16) be the binary sequence corresponding to the concatenation of the 16 chromosomes of the *k*–th strain.

• Phase 3: hierarchical clustering. In the final step we used the clustering package of the scipy scientific library ([54]) to perform a hierarchical clustering of the binary vectors *Ŝ*_1_,*Ŝ*_2_,…,*Ŝ*_39_. The chosen metric is the Hamming distance, while UPGMA is the selected linkage method.

## Competing interests

The authors declare that they have no competing interests.

## Authors’ contributions

GM, RM, NP and RG conceived of the project, GB implemented and tested the algorithm, GM, RM, NP and RG provided guidance for the project, and GM, GB, NP, RG and RM wrote the paper. All authors read and approved the final manuscript.

## Supplementary Material

Additional file 1: Table S1, Statistics of L–grams A table with statistics of *L*–grams as in Methods, for complete yeast strains. Click here for file

Additional file 2: REGENDER performance and complexity The evaluation of regender performance and complexity when compared with several of the most commonly used alignment tools. Click here for file

Additional file 3: Telomeric regions Definition of telomeric regions in RefSeq. Click here for file

Additional file 4: Annotated features Annotations of genomic features for each pme–Ty marked region on RefSeq: two summary tables and the complete.csv table with all Ty, solo–LTR and GRm annotated features. Click here for file

Additional file 5: PME–Ty annotations table A.csv table with 77 loci of pme–Ty in the 39 strains, together with the corresponding features in RefSeq, and the Boolean matrix used to classify such segments as either almost–conserved or fully non–conserved. Click here for file
