# Supplementary figures and images for: Mobilomics in Saccharomyces cerevisiae strains
Source: BMC Bioinformatics. 2013 Mar 20;14:102. doi: 10.1186/1471-2105-14-102 (PMC3684551; doi:10.1186/1471-2105-14-102)

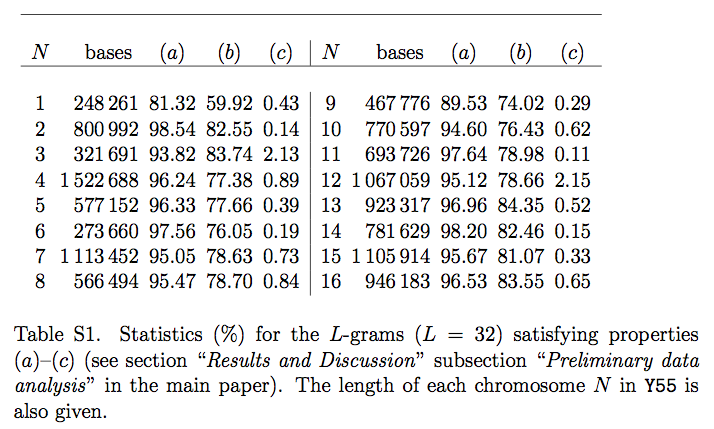

Supplement: Additional file 1: Table S1, Statistics of L–grams — A table with statistics of L–grams as in Methods, for complete yeast strains. [file 1471-2105-14-102-S1.tiff]

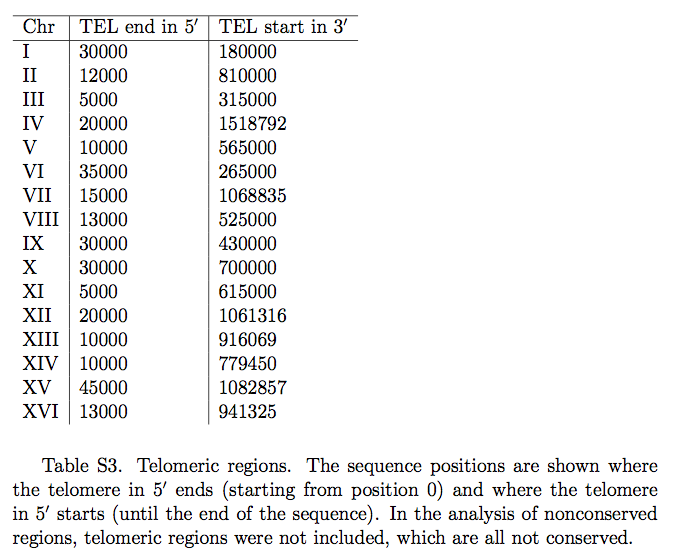

Supplement: Additional file 3: Telomeric regions — Definition of telomeric regions in RefSeq. [file 1471-2105-14-102-S3.tiff]

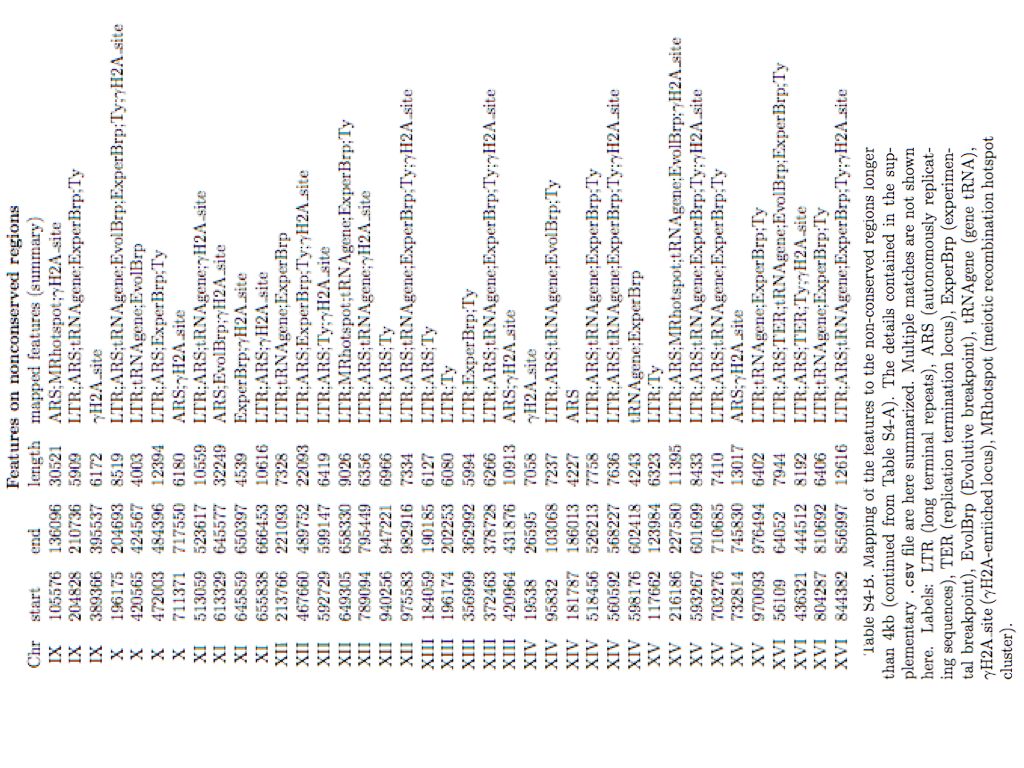

Supplement: Additional file 4: Annotated features — Annotations of genomic features for each pme–Ty marked region on RefSeq: two summary tables and the complete.csv table with all Ty, solo–LTR and GRm annotated features. [file 1471-2105-14-102-S4.zip › TableS4-A.tiff]

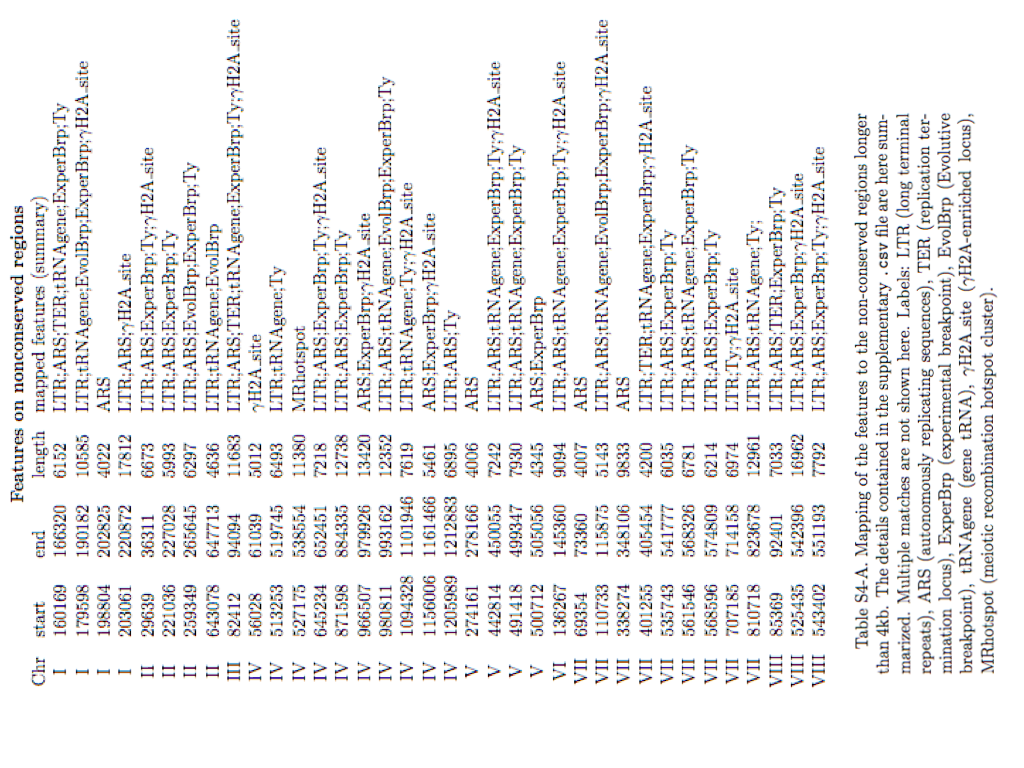

Supplement: Additional file 4: Annotated features — Annotations of genomic features for each pme–Ty marked region on RefSeq: two summary tables and the complete.csv table with all Ty, solo–LTR and GRm annotated features. [file 1471-2105-14-102-S4.zip › TableS4-B.tiff]
