# Supplementary material for: Mobilomics in Saccharomyces cerevisiae strains
Source: BMC Bioinformatics. 2013 Mar 20;14:102. doi: 10.1186/1471-2105-14-102 (PMC3684551; doi:10.1186/1471-2105-14-102)
Supplement: Additional file 2: REGENDER performance and complexity — The evaluation of regender performance and complexity when compared with several of the most commonly used alignment tools. [file 1471-2105-14-102-S2.pdf]

Additional material
Mobilomics in *S. cerevisiæ* strains

Giulia Menconi [1], Giovanni Battaglia [2],
Roberto Grossi [2], Nadia Pisanti [2] and Roberto Marangoni [2,3]

[1]Istituto Nazionale di Alta Matematica, Città Universitaria, 00185 Roma, Italia

[2]Dipartimento di Informatica, Università di Pisa, 56127 Pisa, Italia.

[3]CNR-Istituto di Biofisica, 56124 Pisa, Italia.

REGENDER performance and complexity

The evaluation of REGENDER performance and complexity has been discussed in the Master thesis "Strumenti computazionali per la mobilomica, una nuova branca della bioinformatica" by Emiliano Biscardi, presented at the University of Pisa, in March 2011, and publicly available at the URL: `http://etd.adm.unipi.it/theses/available/etd-02202011-152231`. In this work, Biscardi compared REGENDER with several of the most commonly used alignment tools. A quantitative comparison concerning the space and time complexity has been measured in on the given dataset. A qualitative comparison concerning the location and the length of the conserved sequences detected by each tool has been also performed. Both kinds of comparison are summarized in the enclosed table. The comparison of the conserved regions detected is shown in the enclosed figure, where the case of ChrIV of `RefSeq` and `Y55` is presented. The experimented tools were: `AVID` [2], `BLAST`[1], `GSALIGN`[10], `LAGAN` [3], `LASTZ` [6], `MGA` [7], `MUMMER` [4, 5, 8], `MURASAKI` [9].

References

[1] Stephen F. Altschul, Warren Gish, Webb Miller, Eugene W. Myers, and David J. Lipman. Basic local alignment search tool. *Journal of Molecular Biology*, 215(3):403–410, 1990.

[2] Nick Bray, Inna Dubchak, and Lior Pachter. Avid: A global alignment program. *Genome Research*, 13:97–102, 2003.

Tool	Time	Resident memory	Conserved regions	Shared coverage
AVID	+ 764.09 %	+ 54.88 %	+ 0.99 %	98.64 %
BLAST	- 60.94 %	- 89.29 %	- 2.18 %	96.4 %
GSALIGN	+ 477.90 %	- 47.12 %	- 17.74 %	80.37 %
LAGAN	+ 995.30 %	+ 272.15 %	+ 0.87 %	98.30 %
LASTZ	+ 244.75 %	- 56.04 %	- 15.74 %	82.54 %
MGA	- 2.76 %	- 94.06 %	- 64.97 %	34.82 %
MUMMER	- 56.63 %	- 91.20 %	- 17.69 %	81.37 %
MURASAKI	+ 275.41 %	+ 1035.87 %	- 25.59 %	70.12 %

Table 1: **Comparison between REGENDER and the most used alignement tools**. For each tool, it is shown the percentage of the average variation of the following parameters, when compared to the corresponding ones of REGENDER: execution time, allocated resident memory, conserved regions found, shared aligned bases.

[3] Michael Brudno, Choung B. Do, Gregory M. Cooper, Michael F. Kim, Eugene Davydov, NISC Comparative Sequencing Program, Eric D. Green, Arend Sidow, and Serafim Batzoglou. Lagan and multi-lagan: Efficient tools for large-scale multiple alignment of genomic dna. *Genome Research*, 13:721–731, 2003.

[4] Arthur L. Delcher, Simon Kasif, Robert D. Fleischmann, Jeremy Peterson, Owen White, and Steven L. Salzberg. Alignment oh whole genomes. *Nucleic Acids Research*, 27(11):2369–2376, 1999.

[5] Arthur L. Delcher, Adam Phillippy, Jane Carlton, and Steven L. Salzberg. Fast algorithms for large-scale genome alignment and comparison. *Nucleic Acids Research*, 30(11):2478–2483, 2002.

[6] Robert S. Harris. *IMPROVED PAIRWISE ALIGNMENT OF GENOMIC DNA*. PhD thesis, The Pennsylvania State University - The Graduate School - College of Engineering, 2007.

[7] Michael Höhl, Stefan Kurtz, and Enno Ohlebusch. Efficient multiple genome alignment. *Bioinformatics*, 18 Suppl.1:S312–S320, 2002.

[8] Stefan Kurtz, Adam Phillippy, Arthur L. Delcher, Michael Smoot, Martin Shumway, Corina Antonescu, and Steven L. Salzberg. Versatile and open software for comparing large genomes. *Genome Biology*, 5:R12, 2004.

[9] Kris Popendorf, Hachiya Tsuyoshi, Yasunori Osana, and Yasubumi Sakakibara. Murasaki: A fast, parallelizable algorithm to find anchors from multiple genomes. *PLoS ONE*, 5(9):e12651, 2010.

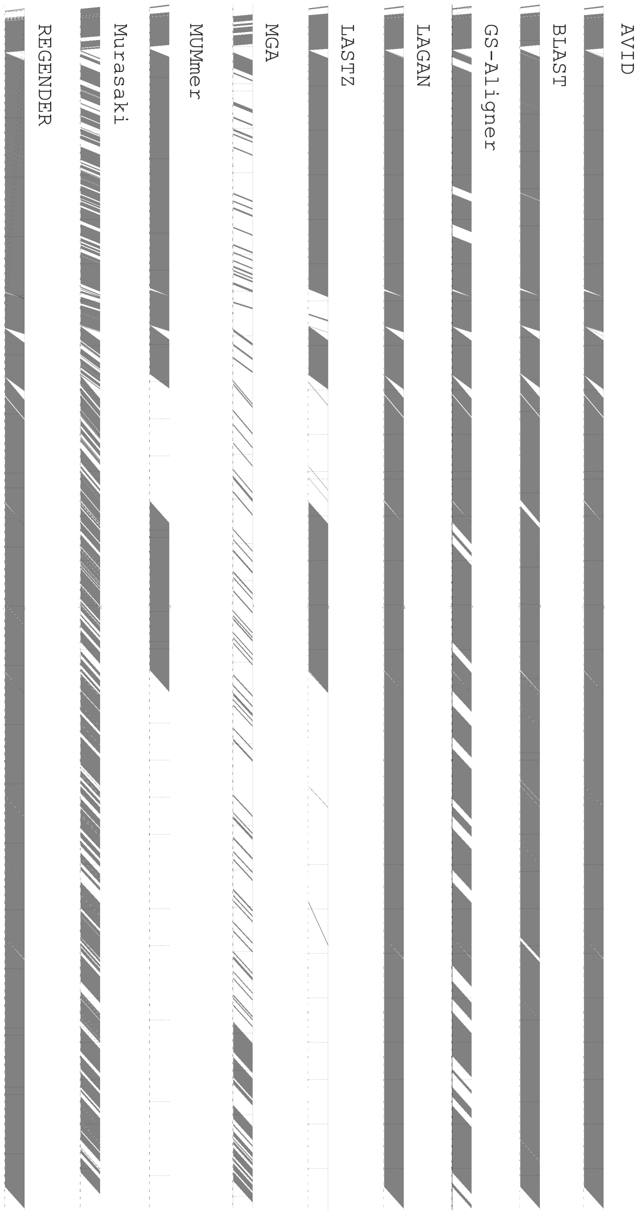

Figure 1: **Comparison of identified conserved regions.** All the plots refer to ChrIV in `RefSeq` (top) and in `Y55` (bottom). The shadowed regions are detected as conserved by the tool indicated in the top line.

[10] Arthur Chun-Chieh Shih and Wen-Hsiung Li. Gs-aligner: A novel tool for aligning genomic sequences using bit-level operations. *Molecular Biology And Evolution*, 20(8):1299–1309, 2003.
